# Supplementary material for: “I’m tired of black boxes!”: A systematic comparison of faculty well-being and need satisfaction before and during the COVID-19 crisis
Source: PLoS One. 2022 Oct 6;17(10):e0272738. doi: 10.1371/journal.pone.0272738 (PMC9536586; doi:10.1371/journal.pone.0272738)
Supplement: S1 Table — Data of Sample 1 (face-to-face teaching) was obtained before and data of Sample 2 (online teaching) during the COVID-19 pandemic. Participants were instructed to answer the session-specific questionnaire 3 to 6 times. (DOCX) [file pone.0272738.s001.docx]

**S1 Table. Data structure of the answered basic and session-specific questionnaires**

|  | | Sample 1 | | Sample 2 | |
| --- | --- | --- | --- | --- | --- |
|  | | *n* | % | *n* | % |
| Participants completed … | |  |  |  |  |
| Basic questionnaire only (excluded from analyses) | | 6 | – | 63 | – |
| Basic questionnaire and session-specific questionnaire | | 89 | 88.1 | 60 | 84.5 |
| Session-specific questionnaire only | | 12 | 11.9 | 11 | 15.5 |
| Final sample size | | 101 |  | 71 |  |
| Frequency of answered session-specific questionnaires | |  |  |  |  |
| 1 |  | 3 | 3.0 | 19 | 26.8 |
| 2 |  | 6 | 5.9 | 8 | 11.3 |
| 3 |  | 7 | 6.9 | 6 | 8.5 |
| 4 |  | 17 | 16.8 | 11 | 15.5 |
| 5 |  | 68 | 67.3 | 14 | 19.7 |
| 6 |  | – | – | 12 | 16.9 |
| 10 |  | – | – | 1 | 1.4 |
| Data of Sample 1 (face-to-face teaching) was obtained before and data of Sample 2 (online teaching) during the COVID-19 pandemic. Participants were instructed to answer the session-specific questionnaire 3 to 6 times. | | | | | |
