# Supplementary material for: “I’m tired of black boxes!”: A systematic comparison of faculty well-being and need satisfaction before and during the COVID-19 crisis
Source: PLoS One. 2022 Oct 6;17(10):e0272738. doi: 10.1371/journal.pone.0272738 (PMC9536586; doi:10.1371/journal.pone.0272738)
Supplement: S2 Table — AUT = autonomy, COM = competence, REL = relatedness, SE = self-efficacy, TS = teaching satisfaction, STR = stress, TP = technical problems, VS = visible students (on average), ENJ = enjoyment, PRI = pride, BOR = boredom, ANX = anxiety, ANG = anger, SHA = shame. 1–4 are reported as experienced before (B) the pandemic. 5–8 are general emergency online teaching experiences during the pandemic compared (C) against own experiences before the pandemic (Sample 2 only). 9–10 are general (G) experiences during time of data collection. 12–23 are session-specific (S) information and experiences. * p < .05. ** p < .01. *** p < .001. (DOCX) [file pone.0272738.s002.docx]

| **S2 Table. Correlations among all study variables.** | | | | | | | | | | | | | | | | | | | | | |
| --- | --- | --- | --- | --- | --- | --- | --- | --- | --- | --- | --- | --- | --- | --- | --- | --- | --- | --- | --- | --- | --- |
|  | 1 | 2 | 3 | 4 | 5 | 6 | 7 | 8 | 9 | 10 | 11 | 12 | 13 | 14 | 15 | 16 | 17 | 18 | 19 | 20 | 21 |
| 1. AUT−B | – |  |  |  |  |  |  |  |  |  |  |  |  |  |  |  |  |  |  |  |  |
| 2. COM−B | .22^**^ | – |  |  |  |  |  |  |  |  |  |  |  |  |  |  |  |  |  |  |  |
| 3. REL−B | .32^***^ | .32^***^ | – |  |  |  |  |  |  |  |  |  |  |  |  |  |  |  |  |  |  |
| 4. SE−B | .23^**^ | .45^***^ | .09 | – |  |  |  |  |  |  |  |  |  |  |  |  |  |  |  |  |  |
| 5. AUT−C | −.07 | −.02 | .08 | −.11 | – |  |  |  |  |  |  |  |  |  |  |  |  |  |  |  |  |
| 6. COM−C | .10 | .23 | −.04 | .00 | .61^***^ | – |  |  |  |  |  |  |  |  |  |  |  |  |  |  |  |
| 7. REL−C | −.29^*^ | .04 | −.15 | .04 | .45^***^ | .49^***^ | – |  |  |  |  |  |  |  |  |  |  |  |  |  |  |
| 8. TS−C | −.10 | .15 | −.23 | .00 | .54^***^ | .79^***^ | .55^***^ | – |  |  |  |  |  |  |  |  |  |  |  |  |  |
| 9. STR−G | −.21^**^ | −.10 | −.32^***^ | −.03 | −.13 | −.09 | −.12 | −.03 | – |  |  |  |  |  |  |  |  |  |  |  |  |
| 10. TP−G | .14 | −.16 | −.10 | .10 | −.38^**^ | −.31^*^ | −.17 | −.31^*^ | .31^*^ | – |  |  |  |  |  |  |  |  |  |  |  |
| 11. VS−S | −.08 | −.02 | .08 | .03 | .02 | −.23 | .08 | −.14 | −.16 | −.20 | – |  |  |  |  |  |  |  |  |  |  |
| 12. ENJ−S | .10 | −.04 | .03 | .05 | .11 | −.06 | .33^*^ | −.08 | −.12 | −.09 | .28^*^ | – |  |  |  |  |  |  |  |  |  |
| 13. PRI−S | .00 | −.03 | .04 | .07 | .20 | .25 | .51^***^ | .20 | .00 | −.12 | .09 | .41^***^ | – |  |  |  |  |  |  |  |  |
| 14. BOR−S | −.09 | −.14 | .03 | −.03 | .12 | −.03 | −.05 | .02 | −.11 | −.12 | −.14 | −.26^***^ | .00 | – |  |  |  |  |  |  |  |
| 15. ANX−S | −.15 | −.20^*^ | −.22^**^ | −.12 | −.08 | −.12 | .16 | −.09 | .16 | .20 | −.08 | −.15 | .10 | .24^**^ | – |  |  |  |  |  |  |
| 16. ANG−S | .02 | −.07 | −.03 | −.09 | −.03 | .00 | −.13 | −.10 | .05 | .04 | −.11 | −.38^***^ | .05 | .39^***^ | .31^***^ | – |  |  |  |  |  |
| 17. SHA−S | −.15 | −.14 | −.16 | −.11 | .03 | −.13 | .14 | −.07 | .17^*^ | .15 | −.07 | −.20^**^ | .04 | .23^**^ | .79^***^ | .37^***^ | – |  |  |  |  |
| 18. AUT−S | .32^***^ | .05 | .15 | .15 | .25 | −.02 | .09 | −.15 | −.21^*^ | −.04 | .17 | .54^***^ | .19^*^ | −.11 | −.29^***^ | −.32^***^ | −.26^***^ | – |  |  |  |
| 19. COM−S | .20^*^ | .30^***^ | .10 | .32^***^ | .25 | .20 | .21 | .20 | −.13 | −.15 | .16 | .53^***^ | .29^***^ | −.16^*^ | −.46^***^ | −.28^***^ | −.51^***^ | .66^***^ | – |  |  |
| 20. REL−S | .11 | .05 | .05 | .16^*^ | .13 | .12 | .43^***^ | .16 | −.01 | −.17 | .26^*^ | .58^***^ | .45^***^ | −.21^**^ | −.08 | −.34^***^ | −.19^*^ | .34^***^ | .47^***^ | – |  |
| 21. TS−S | .04 | .19^*^ | .01 | .18^*^ | .16 | .12 | .31^*^ | .18 | −.17^*^ | −.14 | .26^*^ | .68^***^ | .40^***^ | −.26^***^ | −.34^***^ | −.42^***^ | −.39^***^ | .53^***^ | .74^***^ | .53^***^ | – |
| AUT = autonomy, COM = competence, REL = relatedness, SE = self-efficacy, TS = teaching satisfaction, STR = stress, TP = technical problems, VS = visible students (on average), ENJ = enjoyment, PRI = pride, BOR = boredom, ANX = anxiety, ANG = anger, SHA = shame. 1-4 are reported as experienced before (B) the pandemic. 5-8 are general emergency online teaching experiences during the pandemic compared (C) against own experiences before the pandemic (Sample 2 only). 9-10 are general (G) experiences during time of data collection. 12-23 are session-specific (S) information and experiences.  ^*^ p < .05. ^**^ p < .01. ^***^ p < .001. | | | | | | | | | | | | | | | | | | | | | |
