# Supplementary material for: “I’m tired of black boxes!”: A systematic comparison of faculty well-being and need satisfaction before and during the COVID-19 crisis
Source: PLoS One. 2022 Oct 6;17(10):e0272738. doi: 10.1371/journal.pone.0272738 (PMC9536586; doi:10.1371/journal.pone.0272738)
Supplement: Attachment — Submitted filename: comments-PONE-D-22-04848.docx [file pone.0272738.s003.docx]

The authors are suggested to make the following improvement before publication:

1. The authors should streamline the Introduction. To improve readability, the authors may benefit from focusing each paragraph on a single topic that supports the study rationale and ending with a sentence telling the reader what is to be concluded from that paragraph.
2. Please move the following contents to the Introduction section.

The ultimate goal during the onset of the COVID-19 pandemic was to keep up teaching and learning higher education. In many western, well-developed countries (including Germany where the present study was conducted), video-based digital tools were 105 quickly available and allowed to offer synchronous teaching formats, which were, however, compromised by problems with bandwidth, poor audio quality, complex technological handling etc (From line 102 to line 107).

3. Please move the following contents to the Present Study section.

The key goal of the present study was to explore how faculty responded to these sudden shifts to video-supported synchronous online teaching, as compared to the habitual face-to-face teaching on university sites, in terms of their basic need satisfaction and subjective well-being (From line 107 to line 110).

4. From line 113 to line 133, almost all the contents are given by the author. To improve the persuasiveness of the arguments, the author should refer to more literatures to justify the arguments.
5. The authors are encouraged to move the sentence in line 154 and line 155 to the end of that paragraph.
6. Please move the following contents to the “SDT and faculty well-being” section.

There is initial empirical evidence that the satisfaction of one or more of the three basic psychological needs for autonomy, competence, and relatedness was associated with different aspects of subjective well-being, including higher levels of positive emotions,

lower levels of negative emotions, and higher teaching satisfaction; this evidence stems from studies conducted both before and during the pandemic [15,27,29,37,38].

7. Please divide the first paragraph of the Results into two or three paragraphs to improve the readability.
8. The authors should make sure they cite prior work adequately in the Discussion section.
9. The authors should ensure that they do not present results in the Discussion section. For instance, contents from line 337 to line 342 should be moved to Result section.
10. The authors are encouraged to shorten “Limitations and directions for future research and higher education” section.
11. The manuscript may benefit from subdividing the Discussion into topics that the authors wish to discuss.
12. There are many brackets in the manuscript. The authors are encouraged to reduce the use of brackets to improve the readability.
